# EREM: Parameter Estimation and Ancestral Reconstruction by Expectation-Maximization Algorithm for a Probabilistic Model of Genomic Binary Characters Evolution

**DOI:** 10.1155/2010/167408

**Published:** 2010-05-06

**Authors:** Liran Carmel, Yuri I. Wolf, Igor B. Rogozin, Eugene V. Koonin

**Affiliations:** ^1^Department of Genetics, The Alexander Silberman Institute of Life Sciences, Faculty of Science, The Hebrew University of Jerusalem, Edmond J. Safra Campus, Givat Ram, Jerusalem 91904, Israel; ^2^National Center for Biotechnology Information, National Library of Medicine, National Institutes of Health, Bethesda, MD 20894, USA

## Abstract

Evolutionary binary characters are features of species or genes, indicating the absence (value zero) or presence (value one) of some property. Examples include eukaryotic gene architecture (the presence or absence of an intron in a particular locus), gene content, and morphological characters. In many studies, the acquisition of such binary characters is assumed to represent a rare evolutionary event, and consequently, their evolution is analyzed using various flavors of parsimony. However, when gain and loss of the character are not rare enough, a probabilistic analysis becomes essential. Here, we present a comprehensive probabilistic model to describe the evolution of binary characters on a bifurcating phylogenetic tree. A fast software tool, EREM, is provided, using maximum likelihood to estimate the parameters of the model and to reconstruct ancestral states (presence and absence in internal nodes) and events (gain and loss events along branches).

## 1. Introduction

Reconstruction of genome evolution critically depends on underlying evolutionary models. Many such models, whether implicit or explicit, have been proposed, reflecting different approaches to model design [1]. Probabilistic models, which describe the likelihoods of evolutionary events along the branches of a phylogenetic tree, are among the most commonly used. With the accumulation of genomic data and the advances in computational power, these models are becoming increasingly detailed and powerful. However, the models that have been most extensively explored relate to sequence evolution; whereas evolution of binary characters has received much less attention. Here, we propose a general probabilistic model of evolution of binary characters. While originally developed to study the evolution of eukaryotic gene architecture, the model is formulated in general notations and would apply to diverse classes of binary characters. For example, the model can be used to study the evolution of gene content among species, or the evolution of genomic markers, morphological characters, and the like. The model assumes that the events along a particular branch at a given site depend on the properties of both the site and the branch. Moreover, the model can accommodate rate variability between sites. A fast and flexible software implementation is offered that can be used to analyze any submodel of the general model. In order to estimate the parameters of the model and to reconstruct ancestral states, an efficient expectation-maximization algorithm, which allows for missing data in the input, was developed. The software can also be used in a simulative mode, generating simulated input data.

## 2. The Model of Evolution

For the sake of concreteness, we present the model in terms of gene architecture, so the binary characters designate the presence or absence of an intron in a particular locus. However, with trivial notational changes, the model is equally applicable for describing evolution of other types of binary characters.

**Figure 1:**
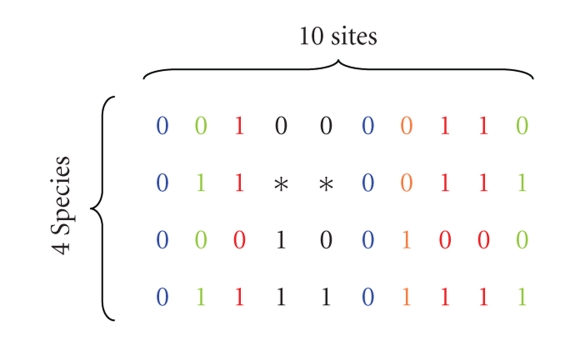
A fragment of a hypothetical input for a 4-species analysis. This is a segment of a multiple alignment of 4 orthologous genes. Notice the gap in one of the genes, designated by * for unknown character. Among the 10 sites in this alignment fragment, there are 6 unique patterns; *ω*_1_ = (0,0,0,0)^*T*^, *ω*_2_ = (0,1,0,1)^*T*^, *ω*_3_ = (1,1,0,1)^*T*^, *ω*_4_ = (0,0,1,1)^*T*^, *ω*_5_ = (0,∗,1,1)^*T*^, and  *ω*_6_ = (0,∗,0,1)^*T*^. This portion of the alignment, therefore, contains 2 copies of *ω*_1_, 2 copies of *ω*_2_, 3 copies of *ω*_3_, and one copy each from *ω*_4_, *ω*_5_, and *ω*_6_.

**Figure 2:**
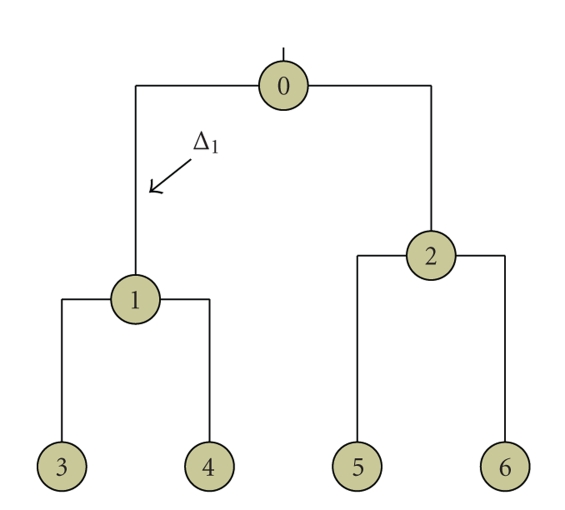
A bifurcating tree with 4 terminal nodes, and 3 internal nodes. Branches are numbered by the node into which they lead.

 A length *n*_*g*_ multiple alignment of a gene across *S* species can be described by a matrix of size *S* × *n*_*g*_. For analysis of binary characters, each such matrix is defined on the alphabet {0,1, *}, with a star (*) indicating a missing value. Each column represents the presence or absence of the binary character at this site (Figure 1). For *G* genes aligned for the same set of species, we have a collection of *G* matrices. Any column in one of the multiple alignments is a vector of length *S* and is called a *pattern*. Let *T* be the phylogenetic tree whose tips match these *S* species, and let us index its nodes by 0,1,…, 2*S* − 2, with the convention that 0 is the index of the root. We use the same indexing for the branches, with the convention that branch *i* leads into node *i* (Figure 2). We assume that the topology of *T*, as well as all branch lengths, Δ_1_,…, Δ_2*S*−2_, is known.

 Let *q*_*t*_ denote the state of node *t* (i.e., 0 or 1), and let *q*_*t*_^*P*^ be the state of its parent node. We denote by *T*(*g*, *t*) the transition matrix, such that *T*_*ij*_(*g*, *t*) = Pr(*q*_*t*_ = *j* | *q*_*t*_^*P*^ = *i*, *g*) is the probability of finding node *t* in state *j* given that its parent node is in state *i*, and given that we are looking at gene *g*. The evolutionary model assumes

(1) $$T\left(g,t\right)=\left(\begin{array}{cc} 1-\xi_{t}\left(1-e^{-\eta_{g}\Delta_{t}}\right) & \xi_{t}\left(1-e^{-\eta_{g}\Delta_{t}}\right)\\ 1-\left(1-\phi_{t}\right)e^{-\theta_{g}\Delta_{t}} & \left(1-\phi_{t}\right)e^{-\theta_{g}\Delta_{t}} \end{array}\right).$$

Here, *ξ*_*t*_ and *ϕ*_*t*_ are the intron gain and loss coefficients of branch *t*, and *η*_*g*_ and *θ*_*g*_ are the intron gain and loss rates of gene *g*. Clearly, the overall probability of gain, loss, or retention of an intron in gene *g* along branch *t* depends on both the particular gene and the particular branch. For instance, the probability of an intron to be gained in gene *g* along branch *t* includes a contribution of the branch (*ξ*_*t*_), and a contribution of the gene (1 − *e* ^−*η*_*g*_Δ_*t*_^). To complete the probabilistic model on the phylogenetic tree, we define *π*_0_ as the probability of the root to be in state 0 (i.e., to be lacking an intron) at a particular site.

 Note that, in the absence of the branch-specific coefficients (*ξ*_*t*_ = 1 and *ϕ*_*t*_ = 0), the transition matrix defines a two-state continuous-time Markovian process. Such a model is popular in evolutionary studies and is implemented in widely used software, such as PAML [2], PHYLIP [3], and PAUP* [4]; a number of expectation-maximization algorithms have been designed to analyze similar Markov processes on phylogenetic trees [5–7]. However, these models cannot take into account the combined influences of the branches and the genes and therefore are not applicable under our model.

 In sequence evolution, rate variability among sites is customarily accommodated by introducing a random variable *r* with unit mean, known as the *rate coefficient*. This coefficient is used to scale the time units of the phylogenetic tree for each particular site, reflecting a distinction between fast-evolving sites and slow-evolving ones [8, 9]. We use a similar idea here, but in order to keep the gain and loss processes independent, two independent rate coefficients are introduced, *r* ^*η*^ and *r* ^*θ*^, to scale the gene-specific gain and loss rate parameters, *η*_*g*_ ← *r* ^*η*^ · *η*_*g*_ and *θ*_*g*_ ← *r* ^*θ*^ · *θ*_*g*_. The rate coefficients are assumed to be distributed according to the following distributions:

(2) $$\begin{array}{c} r^{\eta }\sim \nu \delta \left(\eta \right)+\left(1-\nu \right)\Gamma \left(\eta ;\;\lambda_{\eta }\right),\\ r^{\theta }\sim \Gamma \left(\theta ;\lambda_{\theta }\right). \end{array}$$

Here, Γ(*x*; *λ*) is the unit-mean gamma distribution of variable *x* with shape parameter *λ*, *δ*(*x*) is the Dirac delta function, and *ν* is the fraction of sites that are incapable of gaining the character and are denoted *zero sites*.

 We have developed an expectation-maximization algorithm to find the maximum likelihood estimators of the model parameters [10, 11]. The full model is described by 2*G* + 4*S* parameters. This number might become exceedingly high and results in high variance to the estimates. To circumvent this problem we have developed a two-phase analysis procedure [12, 13]. In the first, *homogeneous *phase, the genes are assumed to have the same gain and loss rates, thus they are all treated as one concatenated supergene. This sets *G* = 1, reducing the number of parameters to 2 + 4*S*. In the second, *heterogeneous* phase, all the parameters estimated in the homogeneous phase are frozen, and only the gene-specific parameters are estimated. Extensive simulations had proved the effectiveness of this procedure (see below).

 As indicated above, we used this model to study the evolution of the intron-exon gene architecture in eukaryotes. In this case, a coding nucleotide position is marked with 1 if an intron got inserted into the sequence right after it. We generated a set of *G* = 391 orthologous genes upon which we marked the presence or absence of the introns. We ran the model upon this data and computed intron gain and loss coefficients for the different lineages [12], and intron gain and loss rates of the different genes [13].

## 3. The Software Tool

The software, named EREM after Evolutionary Reconstruction by Expectation-Maximization, was written in C++ using Microsoft Visual Studio.NET 2003, and is available from ftp://ftp.ncbi.nlm.nih.gov/pub/koonin/erem/; or http://carmelab.huji.ac.il/software.html. In the web site we provide the source code in both Windows and Unix versions (the latter version is courtesy of Stajich et al. [14]), a compiled executable tested under Windows XP, and full documentation. EREM is designed to perform three different tasks. First, based on the input data, it can estimate the parameters of the model by likelihood maximization. Second, it can reconstruct ancestral states and events along the phylogenetic tree. Third, one can run the software in a simulation mode, where the phylogenetic tree and/or the input data matrices are randomly generated.

 To empirically assess the speed of EREM on a large data set, we performed simulations on a 19-species tree with genes containing a total of 10^5^, 3 · 10^5^, or 5 · 10^5^ sites. On a Pentium 3 GHz machine, this took, on average, 4.2, 9.6, and 12.3 minutes, respectively.

## 4. Parameter Estimation

The maximum likelihood estimation of the model parameters is computed by an expectation-maximization algorithm [10, 11]. This algorithm is a general iterative scheme that guarantees an increase in the likelihood at the end of each iteration. Every iteration is built of two computational procedures, called the expectation step (E-step) and the maximization step (M-step). Briefly, the E-step in our case is carried out using a series of recursions along the phylogenetic tree. In the M-step, the auxiliary function computed at the E-step is maximized in a parameter-by-parameter fashion, using low-tolerance one-dimensional maximization procedures.

 For the full model, some combinations of parameters form invariants (yielding the same ancestral reconstructions), thus the values estimated for some individual parameters are hard to interpret. The fundamental output to be analyzed is therefore the ancestral reconstruction (see next section) and not the estimated values of the model parameters.

## 5. Ancestral Reconstruction

Given the model parameters, EREM computes two types of ancestral reconstructions: (a) the average occupancy fraction (average number of sites with state 1 in any particular gene and in any particular node); (b) the average number of character gains, losses, or retentions that occurred for each gene along each branch. Simulations have shown that the reconstructions are highly accurate. Applied to an intron-exon data set, we obtain a relative error of 1%, 3%, and 11% in estimating the number of introns in internal nodes, the number of loss events along each branch, and the number of gain events along each branch, respectively [12].

EREM can output both a detailed gene-by-gene ancestral reconstruction report, as well as an overall report, summing the results of all the individual genes.

## 6. Simulation Mode

One can run EREM in a simulation mode, in which it can randomly generate any of the two types of inputs: (a) a phylogenetic tree, the user only provides the number of terminal nodes and the time span of the tree; (b) alignment matrices for any number of genes. Here, a model is either provided by the user or is randomly generated. Then, it is used to simulate evolution along the given or simulated phylogenetic tree.

## 7. Input Files

EREM requires an input file which has the form of a formatted text file. The website contains a full description, with examples, of this file and all other files discussed here. If the phylogenetic tree is not simulated, the user should provide it (see the web site for the acceptable formats). 

 If the set of alignment matrices is not simulated, it should be supplied by the user in the form of two text files. The first file lists all the unique patterns in the data, and the second file lists, for each gene, the counts of each of the unique patterns.

## 8. Output Files

The primary output of EREM is a formatted text file that summarizes the input, the values of the estimated parameters, and the overall ancestral reconstruction. Also, for each estimated parameter, EREM produces a history file, which records how the value of this parameter changed along the iterations.

 If a detailed, gene-by-gene ancestral reconstruction was requested by the user, EREM outputs a special text file providing this information. Additionally, if the phylogenetic tree was simulated, the information about it is kept in another text file. If the input data were simulated, the two summary files describing it (see previous section) are stored as text files. In this simulation mode, the user can also request to store the actual state of each site in each node of the tree, for the purposes of comparison with the ancestral reconstruction computed by EREM.

## 9. Matlab Auxiliary Functions

We use Matlab as the chief tool to analyze the results. Consequently, we have written a number of Matlab functions (written for Matlab version R2006a) to facilitate the interaction between Matlab users and EREM. The set of functions can be downloaded from the web site. In particular, these functions allow a Matlab user to generate input files, to read output files, to analyze the ancestral reconstructions, to visualize some of the results, and to compute error bars to the estimations. We emphasize that these utilities are provided in our website to facilitate analysis of EREM output and are not an integral or necessary part of EREM.

## 10. Availability and Requirements

The website (ftp://ftp.ncbi.nlm.nih.gov/pub/koonin/erem/ or http://carmelab.huji.ac.il/software.html) contains detailed description of the formats of all input and output files, as well as a number of examples. Also, a full description of all Matlab auxiliary functions is provided.

 The web site includes an executable for Windows XP Professional, as well as C++ source code in Microsoft Visual Studio.NET 2003 and in Unix (the latter version is courtesy of Stajich et al. [14]).
